# Effects of transcranial pulse stimulation on autism spectrum disorder: a double-blind, randomized, sham-controlled trial

**DOI:** 10.1093/braincomms/fcad226

**Published:** 2023-08-18

**Authors:** Teris Cheung, Tim Man Ho Li, Joyce Yuen Ting Lam, Kwan Hin Fong, Lok Yi Chiu, Yuen Shan Ho, Andy Choi-Yeung Tse, Cheng-Ta Li, Calvin Pak-Wing Cheng, Roland Beisteiner

**Affiliations:** School of Nursing, The Hong Kong Polytechnic University, Hong Kong SAR, China; The Mental Health Research Centre, The Hong Kong Polytechnic University, Hong Kong SAR, China; Department of Psychiatry, The Chinese University of Hong Kong, Hong Kong SAR, China; School of Nursing, The Hong Kong Polytechnic University, Hong Kong SAR, China; School of Nursing, The Hong Kong Polytechnic University, Hong Kong SAR, China; School of Nursing, The Hong Kong Polytechnic University, Hong Kong SAR, China; School of Nursing, The Hong Kong Polytechnic University, Hong Kong SAR, China; Department of Health and Physical Education, The Education University of Hong Kong, Hong Kong SAR, China; Department of Psychiatry, School of Medicine, National Yang Ming Chiao Tung University, Taipei 112, Taiwan; Department of Psychiatry, The University of Hong Kong, Hong Kong SAR, China; Department of Neurology, Medical University of Vienna, Wien 1090, Austria

**Keywords:** transcranial pulse stimulation, randomized controlled trial, autism spectrum disorder, neuromodulation, adolescents

## Abstract

Transcranial pulse stimulation has been proven effective to improve cognition, memory and depressive symptoms of Alzheimer’s disease, but supporting evidence on other neurological diseases or neuropsychiatric disorders remains limited. This study aimed to investigate the effects of transcranial pulse stimulation on the right temporoparietal junction, which is a key node for social cognition for autism spectrum disorder, and to examine the association between transcranial pulse stimulation and executive and social functions. This double-blinded, randomized, sham-controlled trial included 32 participants (27 males), aged 12–17 years with autism spectrum disorder. All eligible participants were randomized into either the verum or sham transcranial pulse stimulation group, on a 1:1 ratio, based on the Childhood Autism Rating Scale screening score. Sixteen participants received six verum transcranial pulse stimulation sessions (energy level: 0.2–0.25 mJ/mm^2^; pulse frequency: 2.5–4.0 Hz, 800 pulse/session) in 2 weeks on alternate days. The remaining 16 participants received sham transcranial pulse stimulation. The primary outcome measure included Childhood Autism Rating Scale score changes, evaluated by parents, from baseline to 3-month follow-ups. Secondary outcomes included a self-reported questionnaire responded to by parents and cognitive tests responded to by participants. A licensed mental health professional evaluated clinical global impression severity, improvement, efficacy and total score. Results revealed significant interactions in Childhood Autism Rating Scale and other secondary outcomes. Significant group and time effects were found in most secondary outcomes. Additionally, significant differences were found between the transcranial pulse stimulation and sham transcranial pulse stimulation groups in Childhood Autism Rating Scale and clinical global impression improvement and total score immediately after 2 weeks of transcranial pulse stimulation intervention (all *P* < 0.05), and effects were sustainable at 1- and 3-month follow-up, compared with baseline. The effect size of Childhood Autism Rating Scale (*d* = 0.83–0.95) and clinical global impression improvement (*d* = 4.12–4.37) were large to medium immediately after intervention and sustained at 1-month post-stimulation; however, the effects were reduced to small at 3-month post-stimulation (*d* = 2.31). These findings indicated that transcranial pulse stimulation over right temporoparietal junction was effective to reduce the core symptoms of autism spectrum disorder, as evidenced by a 24% reduction in the total Childhood Autism Rating Scale score in the verum transcranial pulse stimulation group. Additionally, the clinical global impression total score was reduced by 53.7% in the verum transcranial pulse stimulation group at a 3-month follow-up, compared with the baseline. Participants in the verum transcranial pulse stimulation group had shown substantial improvement at 1- and 3-month follow-ups, compared with baseline, although some of the neuropsychological test results were deemed statistically insignificant. Future replication of this study should include a larger sample derived from multi-nations to determine transcranial pulse stimulation as an alternative top-on treatment option in neuropsychiatry.

## Teaser

This study aimed to investigate the effects of transcranial pulse stimulation on autism spectrum disorder. Cheung *et al*. reported that there was a 24% reduction in the total Childhood Autism Rating Scale score in the transcranial pulse stimulation group after brain stimulation. Transcranial pulse stimulation could be considered as an adjunct treatment option for neurodevelopmental disorders.

## Graphical Abstract

**Graphical Abstract:**
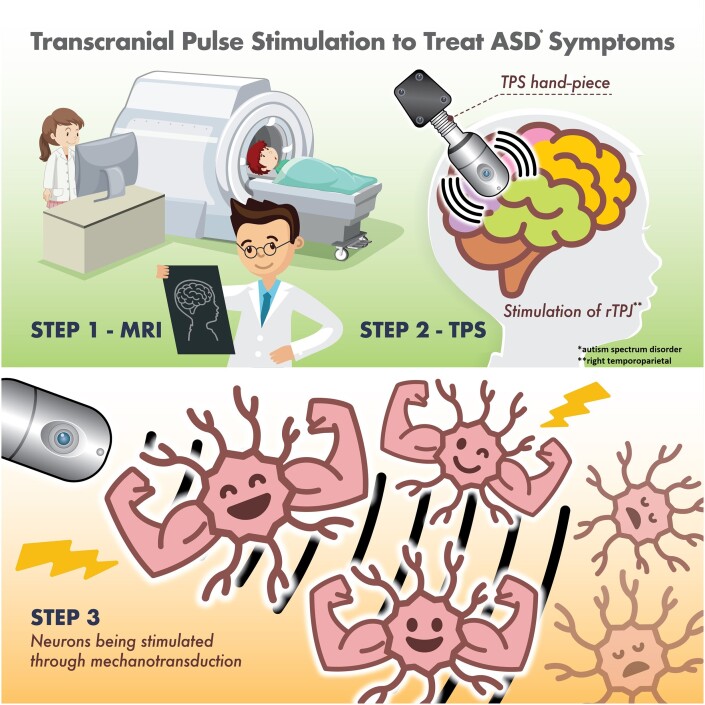

## Introduction

Autistic spectrum disorder (ASD) is a neurodevelopmental disorder characterized by impaired reciprocal social interaction, language dysfunction and restricted interests associated with behaviour problems. A recent systematic review (*N* = 30 212 757) reported that the global prevalence of ASD was 0.6% [95% confidence interval (CI): 0.4–1%]. Subgroup analyses indicated a 0.4% ASD prevalence in Asia (95% CI: 0.1–1) and 1% in America (95% CI: 0.8–1.1).^1^ Further, ASD has been linked with functional impairment and increased risk of psychiatric and medical morbidity.^2^ Furthermore, ASD causes significant distress and mental health–related problems to caregivers.^3^ These negative detrimental effects will inevitably increase the global disease burden and health costs on the health care system, thereby pressing the need to formulate evidenced-based, robust interventional studies to restore optimal well-being in this at-risk, neurodivergent population.

Meanwhile, the exact underlying pathophysiology of ASD remains unknown. Effective and specific pharmacological treatment for core symptoms of ASD remains unavailable.^4^ The mainstream treatment for ASD consists of behavioural intervention and social skill training. However, these treatment options are time-consuming and labour-intensive. Therefore, a new treatment option that is effective and well tolerated by children with ASD is warranted.

### Biological mechanism of TPS

Mechanotransduction is the basic mechanism of transcranial pulse stimulation (TPS). TPS can promote new blood vessel formation (angiogenesis) and nerve regeneration, stimulate vascular growth factors^5,6^ and brain-derived neurotrophic factor^7^ and improve cerebral blood flow. Mechanotransduction is a biological pathway through which the cells convert the mechanical TPS stimulus into biochemical responses, thereby triggering some fundamental cell functions, such as migration, proliferation, differentiation and apoptosis.^8,9^ TPS can stimulate deep cerebral regions (i.e. 8 cm) into the brain. The ultrashort ultrasound pulse could enhance cell proliferation and differentiation in cultured neural stem cells, which plays an important role in brain function repair in central nervous system diseases.^10^ TPS may affect neurons and induce neuroplastic effects, which increase cell permeability,^10^ stimulate mechanosensitive ion channels^8^ and release nitric oxide that causes vasodilation, increased metabolic activity and angiogenesis.^11^

### Rationale for using TPS in this study

TPS is the latest non-invasive brain stimulation (NIBS) used to stimulate the brain by inducing ultrashort ultrasound waves to the brain treatment region. Existing TPS studies revealed TPS as effective and safe in treating Alzheimer’s disease in Austria^12^ and major depressive disorder^13^ in Hong Kong. However, there is no randomized controlled trial (RCT) that has evaluated the efficacy and safety of TPS on young adolescents with ASD thus far. This research gap presents the impetus to execute this trial.

## Materials and methods

The full protocol of this clinical trial was published elsewhere.^14^ This clinical trial was registered with ClinicalTrials.gov (NCT05408793) on 7 June 2022.

### Participants

#### Inclusion/exclusion criteria

Participants were recruited using a QR code flyer embedded with a Qualtrics online application form. This QR code flyer was flagged up in communal areas on university campuses including the Hong Kong Polytechnic University, the University of Hong Kong and the Educational University of Hong Kong. Further, this study was promoted via Hip Hong Society, which is a key local non-governmental organization for ASD. Inclusion criteria included (i) 12–17 years of age, (ii) Chinese ethnicity, (iii) ASD diagnosis according to the fifth edition of the Diagnostic and Statistical Manual of Mental Disorders (DSM-5), (iv) no changes in their drug regime in the past 3 months and (v) currently taking prescribed psychotropic medications for ≥3 months.

Exclusion criteria included (i) a fifth edition of the Diagnostic and Statistical Manual of Mental Disorders diagnosis other than ASD, (ii) concomitant major medical conditions, (iii) neurological problems (e.g. brain tumour or brain aneurysm), (iv) or other clotting disorders or thrombosis, (v) a metal implant in the brain/brain-treated region, (vi) corticosteroid treatment in the past 6 weeks before enrolment or (vii) a Childhood Autism Rating Scale (CARS) score of <30 (i.e. no ASD).

All participants’ parents signed written informed consent following the Declaration of Helsinki. Information sheet containing the aims and purpose of this trial, intervention dose, trial duration, anonymity, confidentiality and right of withdrawal was explained to participants and their parents. The Human Participants Ethics Committee of the Hong Kong Polytechnic University approved this study (reference no: HSEARS20220228005). The recruitment period was 1 June to 30 November 2022.

#### Sample size

G*Power 3.1.9.4 was used for sample size calculation. A similar RCT design^15^ on ASD using repeated transcranial magnetic stimulation (rTMS) indicated an effect size of 0.5. Considering our study was the first RCT evaluating the effects of TPS on ASD core symptoms nationwide, thus we took reference to this rTMS trial in the sample size calculation. A sample size of 17 participants per group (34 participants in total) was deemed appropriate to detect the difference with 80% power in this study.^14^ Unfortunately, two eligible participants (one in the verum TPS group and one in the sham TPS group) who undertook pre-TPS MRI were infected with coronavirus disease 2019 (COVID-19) with high fever and other COVID-19-related symptoms that required hospitalization, and parents were apprehensive to join this trial upon participants’ hospital discharge. Additionally, no eligible participants were on the waitlist when the extended recruitment period was over. We decided to go ahead with 32 participants upon consultation with all researchers involved in this project, as it was least likely to affect our effect size and estimated power of this study.

### Aim of the study

This study aimed to evaluate the efficacy and tolerability of TPS on young adolescents with ASD.

### Research hypothesis

The verum TPS group would have at least a 50% reduction rate in autistic severity than the sham TPS group, and effects are sustainable at 2-week post-stimulation and at 1- and 3-month post-stimulation follow-up (F/U), as evaluated by the CARS. The sham TPS demonstrated a <5% reduction in autism severity after 2 weeks of intervention and <3% reduction in autism severity at 1- and 3-month post-stimulation F/Us. Participants in the verum TPS group demonstrated 30% improvement in all the secondary outcomes, including Autism Spectrum Quotient (AQ), Australian Scale for Asperger’s Syndrome (ASAS), Stroop test, Digit Span Test and clinical global impression (CGI) severity, improvement and efficacy scale.

#### Study design

**Figure 1:**
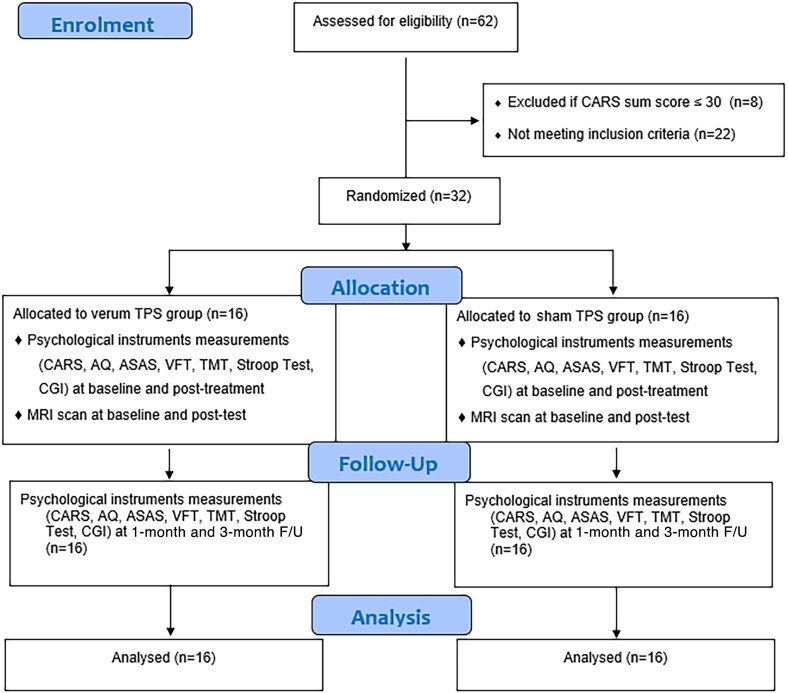
**CONSORT flow diagram for participant enrolment, randomization, allocation and follow-up.** AQ, Autism Spectrum Quotient; ASAS, Australian Scale for Asperger’s Syndrome; CARS, Childhood Autism Rating Scale; CGI, clinical global impression; TMT, Trail Making Test, Stroop test; VFT, Verbal Fluency Test.

This two-armed, randomized, double-blind, sham-controlled trial evaluated the efficacy and tolerability of a 2-week TPS treatment on young adolescents with ASD. This study design strictly complied with the Consolidated Standards of Reporting Trials (CONSORT) statement^16^ and Good Clinical Practice. Participants were randomly allocated into the verum or sham TPS groups on a 1:1 ratio. All the participants’ parents were informed about the randomization procedures that participants had a 50% chance of receiving the verum or sham TPS. This study was conducted following the Declaration of Helsinki.^17^ Both groups were measured at baseline (T_1_), immediately after the 2-week intervention (T_2_), and at 1- (T_3_) and 3-month (T_4_) F/Us (Fig. 1).^18^ All participants’ parents were advised to report any changes in the participants’ prescribed treatment regime during the 1- and 3-month F/U periods. Parents were also advised to avoid adopting other modes of pharmaceutical treatments over the counter without medical prescription during the study period. However, parents reported no changes in medication during the F/U period.

#### Screening of participants’ eligibility

Participants’ parents completed an online application form soliciting socio-demographic information of potential participants [e.g. age, gender, educational background, monthly family household income (HK$), living circumstances, school year, psychiatric history and duration of ASD diagnosis (in years/months), age upon ASD diagnosis, duration of taking prescribed medications (in years/months), current drug regimen, and family history of psychiatric disorder] in Section A. Parents filled in the online screening instrument (CARS), in Section B. Participants with a CARS total score of >30 were recruited (CARS scores of 30–36.5 indicate mild to moderate autism, and scores of 37–60 indicate severe autism).^19^ After the screening procedures, eligible participants’ parents were invited to attend a Zoom interview, chaired by the principal investigator and a research associate. The Zoom interview aimed to affirm and validate the eligibility of each participant and established initial rapport with participants and their parents. Participants’ medical/psychiatric history, medication adherence and neurodevelopmental history were further explored in the Zoom interviews if indicated.

#### Randomization, allocation and masking

This study used block randomization. Each block comprised six participants on a 1:1 ratio between the verum and sham TPS group (total: six blocks). The randomization procedure assigned each participant to a serial reference number generated by a computer. These numbers were decoded until the intervention group was assigned. An offsite statistician, who was an independent team member not involved in the enrolment, intervention or assessments, performed the randomization process. Participants and research assistants who were responsible for baseline and post-test were blinded to the group allocation (Fig. 1). The interventionist was also blinded, and a TPS technician on site was responsible to change the TPS hand-piece distinguishing participants from the verum and sham TPS groups. Participants were requested to guess their treatment allocation in the last TPS session to determine the success of the blinding.^20^

#### Neuroimaging procedures

Participants were asked to change into a gown upon arrival at our UBSN/PolyU. We advised the participant that metal objects were prohibited in the scanner room because participants had to undertake neuroimaging that involved magnets. Participants had to remove any metal jewellery/accessories that might interfere with the MRI scanner. A safe checklist was used to assess all participants’ medical history, especially if they had any metal implant/metallic foreign bodies (e.g. pacemakers, cochlear implants and aneurysms) inside their bodies. This study will exclude participants with claustrophobia as we required their structural image to calibrate into the TPS system. Participants were asked to lie down on the scanning table comfortably and a radiographer would offer earplugs to filter the noise running out from the scanner throughout the procedure, and a call bell was given to the participant to alert the radiographer for any discomfort throughout the neuroimaging procedures. The radiographer would communicate with the participants via the intercom to make sure that they were comfortable throughout the neuroimaging procedures that usually take approximately 45 min. The research associate and the participant’s parents would stay in the MRI venue until the procedures were completed.

#### Treatment setting

The TPS interventions were performed at the Integrative Health Clinic (AG057t), the Hong Kong Polytechnic University. The principal investigator who was a licensed medical staff administered the TPS to participants of both groups.

#### TPS intervention

**Figure 2:**
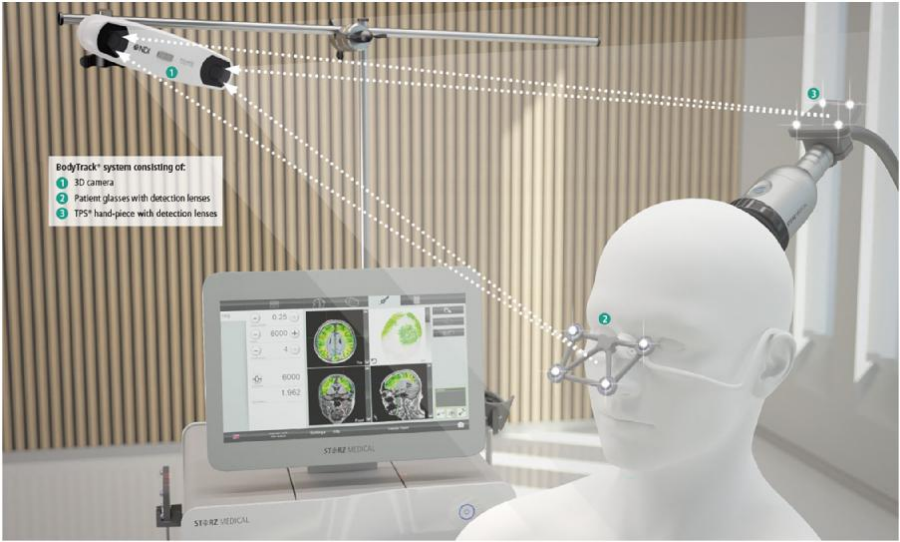
**The TPS system (image source: NEUROLITH, TPS manufacturer).** BodyTrack® system consisting of (1) 3D camera (left top); (2) patient glasses with detection lenses; (3) TPS® hand-piece with detection lenses.

The TPS® system (developed by NEUROLITH, Storz Medical AG, Tägerwilen, Switzerland) consists of a mobile single transducer and an infrared camera system, which incorporates neuro-navigation. This TPS® system can generate a single ultrashort (3 µs) ultrasound shockwave pulses with 0.2–0.25 energy levels (mJ/mm^2^) and 2.0–4 Hz pulse frequencies (pulses per second). The calibration procedures used the MRI T_1_-weighted images of all individual participants. A participant was seated in an adjustable electronic chair and wore a BodyTrack® system that consisted of tracking glasses with detection lenses, a 3D camera and a TPS hand-piece. The interventionist used each participant’s MRI T_1_-weighted images to locate the right temporoparietal junction (rTPJ), and a treatment circle was drawn on the targeted treatment region before the commencement of each TPS session. The tracking system could ensure that participant’s head matched their own MRI T_1_-weighted images, so that the interventionist can visualize each pulse applied and documented in real time. The real-time tracking of the hand-piece position allows automatic visualization of the treated brain region, as highlighted in green on the monitor (Fig. 2).

#### Treatment brain region and intervention dose

**Figure 3:**
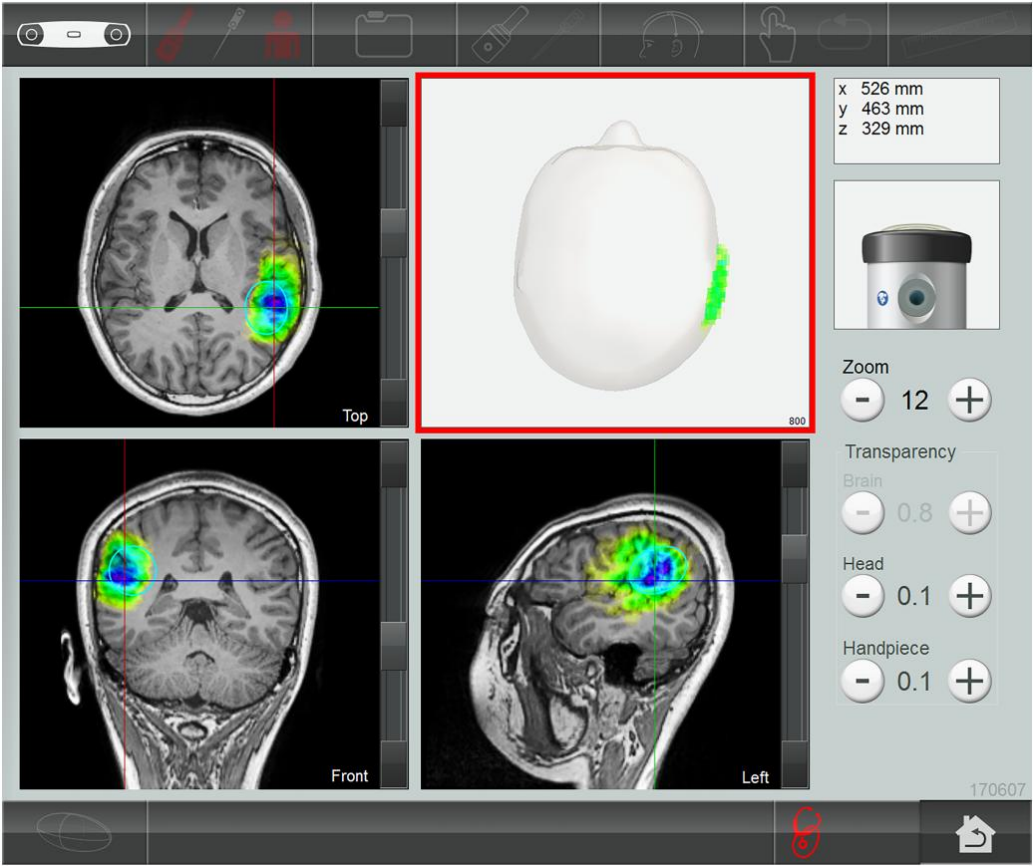
**Participant’s MRI (T_1_-weighted) images.** The stimulated treatment region (rTPJ) after TPS intervention.

Right temporoparietal junction (rTPJ) is a key node for social cognition. This junction was a typical area of difficulty among individuals with ASD.^21^ We selected the treatment brain region based on previous research,^15^ which had demonstrated abnormal brain activation in ASD and can improve social communication in adolescents and young adults with ASD. The first TPS study^12^ nationwide was conducted on 35 adult patients with Alzheimer’s disease, using 6000 TPS pulses (i.e. global stimulation) on each patient in each session throughout the study. The present study delivered 800 pulses (confirmed with the NEUROLITH—TPS manufacturer, neurologist and psychiatrist in the project team) in each TPS session, because our participants were young adolescents aged 12–17 years old, and we only targeted the rTPJ as the treatment region. Each TPS session lasted for approximately 30 min. The interventionist manually moved the TPS hand-piece over the participant’s skull to deliver the TPS pulses in the treatment region with the reference of MRI T_1_-weighted brain images. All TPS treatment sessions were recorded for individual evaluation of the intra-cerebral pulse localizations. Each participant was administered 800 TPS pulses in each TPS session, and thus, the mean pulse per voxelis unavailable. Stimulated area indicating the location and depthness was automatically shown in different colours on the computer screen in real-time navigation of the TPS hand-piece during the TPS procedures (Fig. 3).

#### Sham TPS

The procedure for sham stimulation was identical, except that the silicone oil used in the verum TPS group was replaced by an air-filled stand-off cushion in the hand-piece. This sham device produced similar sounds and stimuli in the participant’s head. All participants/parents were advised to continue with their routine medication regimen throughout the TPS intervention period.

#### Intervention dose

All eligible participants were computerized randomized to receive six verum TPS treatment sessions or sham TPS, with three sessions per week on alternate days for 2 consecutive weeks. The outcome measurements were assessed at baseline, immediately after intervention (2 weeks), and at 1- and 3-month F/Us (see Fig. 1, CONSORT flow diagram).

#### Measures

This study assessed nine outcomes. CARS was the primary outcome, whereas AQ, ASAS, Social Responsiveness Scale (SRS), Trail Making Test (TMT) (1, 2 and 3), Verbal Fluency Test (VFT), Stroop test (1, 2 and 3), Digit Span Test (forward and backward) and CGI (severity, improvement, efficacy and total score) were the secondary outcomes.

#### CARS

CARS is a 15-item behavioural rating scale developed to identify autism and examine its severity. The 15 items include different domains (e.g. relating to people, imitative behaviour, emotional response, body and object use, adaptation to change, visual/listening/perceptive response, fear or anxiety, verbal/non-verbal communication, activity level, consistency of intellective relations and general impressions).^22^ Total scores range from 15 to 60 where scores below 30 indicate a non-autistic range, scores 30–36.5 indicate mild to moderate autism, and scores 37–60 indicate severe autism.^23^ The assessment tool is well validated and has been widely used in various ASD studies.^24–26^ Previous NIBS study^27^ also used CARS scores as their primary outcomes and parents filled out the baseline and post-stimulation CARS score. CARS was assessed at baseline, immediately after post-stimulation at Week 2 and at 1- and 3-month post-stimulation F/U.

#### Secondary outcomes

##### AQ—adolescent version

AQ is a self-report instrument for autistic traits, with scores of 0–50. AQ consisted of 10 questions assessing (i) social skills, (ii) attention switching, (iii) attention to detail, (iv) communication and (v) imagination.^28^

##### SRS

SRS is an instrument measuring the continuum of autism symptom severity, which is commonly used in children and adolescents and aged 4–18 years.^29^ SRS consists of 65 items subsumed in five *a priori* content areas of social deficits, i.e. social awareness, social cognition, social communication, social motivation and autistic mannerisms. Parents rate each item on a 4-point Likert scale (0–4). The higher the scores, the more severe the social deficits. SRS has satisfactory reliability and validity for measuring autism symptoms in individuals aged <18 years.^30,31^

##### ASAS

ASAS is a 25-item instrument used to screen the behaviours and abilities indicative of Asperger’s syndrome in participants older than 6 years old.^32^

We aimed at recruiting a homogenous cohort for this trial, and thus, we deliberately used SRS in addition to CARS to determine autism severity, and we also used ASAS intentionally to determine any participants with Asperger’s syndrome or intellectual disability (i.e. as cross-check measures).

##### TMT (1, 2 and 3)

TMT is divided into two parts. This test is used to test an individual’s speed of attention, sequencing, visual search, mental flexibility and motor function. The TMT scores are expressed in terms of time taken in seconds to complete Tests 1, 2 and 3, including the number of errors in each subtest. The total time spent on TMT-1 and 2 reflects visual searching ability. The total time spent on TMT-3 reflects processing speed and mental tracking. Additionally, the TMT error score reflects working memory (WM) and executive functioning. TMT test has good reliability and validity.^33^

##### VFT

VFT asked participants to produce as many words as possible from a semantic and phonemic category in a given time. The time used in producing these words will be counted in 60 s per category. Participants will be asked to produce three categories (i.e. animals, vegetables and fruits) in this trial. Repetition of the entire word is not allowed. This VFT has good reliability.^34^

##### Stroop test

Stroop test is a neuropsychological test commonly used to assess the inhibition control component of executive function. It tests the participant’s ability to inhibit cognitive interference that occurs when the processing of a specific stimulus feature impedes the simultaneous processing of a second stimulus attribute.^35^

##### Digit Span Test

The Digit Span Test was used to measure the WM of participants (both forward and backward digit recall). The Digit Span Test has been previously used to assess WM in school-aged children with ASD in Hong Kong.^36^ The Digit Span Test (backward) is arguably difficult for all populations, and children are no exception. Hence, we administer this test to assess whether participants’ Digit Span Test score has any difference after the TPS administration in this study. Indeed, some of our participants encountered such difficulty in this test, but we encouraged them to try their best to attempt this test, considering that we aimed to investigate the total duration of completion (in minutes/seconds), and we counted the number of errors in this test for comparison with other outcome measures.

##### CGI severity, improvement, efficacy and total score

CGI is a 7-point clinician rating scale based on observed and reported symptoms, behaviour and function in the past 7 days. The score should reflect the average severity level across the 7 days because ASD symptoms and behaviour can fluctuate over a week. Clinician used CGI to assess the extent to which the participant’s core symptoms improved or worsened compared with before the intervention.^37^

### Statistical analyses

Means and standard deviations (SDs) were presented for the continuous variables, while numbers and percentages were for the categorical variables. The Mann–Whitney U test and chi-squared test were conducted for continuous and categorical variables, respectively, to examine the presence of any group differences in terms of socio-demographics and outcomes between the treatment and sham groups at baseline. The mixed model for repeated measures was used to test the group (between-participants factor), time (within-participants factor) and group × time interaction effects of the CARS scores and other secondary outcomes between the verum and sham TPS groups. The model was adjusted for age and gender. *Post hoc* analysis was performed with Bonferroni correction to investigate the improvements of outcome in pairwise comparisons across various assessment time points for significant interaction effects. The statistical significance was set at *P*-values of <0.05. For significant interaction effects, *post hoc* analysis was also performed with Bonferroni correction to investigate the improvements of the outcomes in pairwise comparisons across various assessment time points. The level of statistical significance was set at *P*-value < 0.05. A Cohen’s *d* effect size for each outcome was calculated, where *d* = 0.2, 0.5 and 0.8 correspond to small, medium and large effect sizes, respectively. All computations were performed using the statistical software R for Windows (R version 4.2.0).

## Results

### Socio-demographic characteristics

This trial included 32 participants (27 males and 5 females), aged 12–17, with a mean age of 13.5 (SD: 2.03) and 12.81 years (SD: 1.83) for the TPS and sham TPS groups, respectively. All socio-demographic variables between participants and their parents in both the TPS and sham TPS groups demonstrated no significant difference. Participants were predominantly males (84%) and evenly distributed in primary and junior/senior high school. Participants had an average of 4.3–4.5 years of having an ASD diagnosis. All participants were currently taking medications for at least 8 years, and half of them (50%, *n* = 16) had good drug compliance. All (except two separated/divorced) parents were married, with <20% having a bachelor’s degree or above. Thirteen parents (41%) had a history of psychiatric disorder, and 59% of them were educated up to secondary school. All socio-demographic variables demonstrated no statistically significant differences between the TPS and sham TPS groups (all *P* > 0.05) (Table 1).

**Table 1:** Demographic characteristics of participants (*n* = 32)

	TPS group	Sham TPS	
	Mean (SD)/*n* (%)	Mean (SD)/*n* (%)	*P*
**Participants**			
Age	13.50 (2.03)	12.81 (1.83)	0.32
Gender			>0.99
Male	13 (81%)	14 (88%)	
Female	3 (19%)	2 (12%)	
Schooling			0.57
Senior primary	6 (37.5%)	9 (56.3%)	
Junior high school	7 (43.8%)	5 (31.3%)	
Senior high school	3 (18.7%)	2 (12.4%)	
Mean years of having ASD diagnosis	4.56 (3.08)	4.27 (2.52)	0.58
Currently on medication			>0.99
Yes	16 (100%)	16 (100%)	
Mean age of starting medication	8.18 (2.40)	7.92 (2.50)	0.72
Duration of taking medication (months)	70.80 (29.20)	55.27 (31.13)	0.46
Drug compliance			0.57
Good	6 (37.5%)	9 (56.2%)	
Fair	4 (25%)	3 (18.8%)	
Poor	6 (37.5%)	4 (25%)	
Family history of psychiatric disorders^a^			0.72
Yes	6 (37.5%)	7 (43.8%)	
No	10 (62.5%)	9 (56.2%)	
**Parents**			
Marital status			0.14
Married	16 (100%)	14 (87.5%)	
Separated/divorced		2 (12.5%)	
Education level			0.52
Primary school or below	1 (6%)	1 (6%)	
Secondary school	8 (50%)	11 (69%)	
Associate degree	4 (25%)	1 (6%)	
Bachelor’s degree or above	3 (19%)	3 (19%)	

ASD, autistic spectrum disorder; SD, standard deviation; TPS, transcranial pulse stimulation.

a First-degree relative, including parents and siblings.

### Effects of the TPS intervention on ASD

Table 2 presented the effects of TPS intervention on the primary and secondary outcome scores. Primary and secondary outcomes at baseline were not statistically significantly different between the TPS and sham TPS groups (all *P* > 0.05). This table reports the group, time and group × time interaction effects of the TPS intervention. We found significant group effects for CGI (improvement) and overall CGI score and significant time effects for most secondary outcomes (except ASAS, Digit Span Test backward and VFT_60 s). Significant interaction effects were observed for the primary outcome (CARS) and other secondary outcomes (Stroop Test 2 and TMTs 2 and 3), CGI severity and, improvement and overall CGI scores.

**Table 2:** Outcome measures of participants at each time point and the effects

Outcomes	TPS group				Sham TPS				Effect			
Time point^a^	T_1_ Mean (SD)	T_2_ Mean (SD)	T_3_ Mean (SD)	T_4_ Mean (SD)	T_1_ Mean (SD)	T_2_ Mean (SD)	T_3_ Mean (SD)	T_4_ Mean (SD)	Baseline diff *P*	Group *P*	Time *P*	Group × time *P*
CARS	30.81 (5.91)	23.44 (5.66)	23.44 (7.12)	23.56 (6.50)	27.94 (7.05)	26.00 (4.59)	26.75 (6.05)	25.88 (5.43)	0.14	0.73	<0.001***	0.02*
AQ	33.12 (5.41)	30.12 (6.15)	28.62 (5.82)	30.31 (6.61)	31.81 (4.66)	30.25 (6.70)	30.44 (6.15)	30.31 (7.34)	0.48	0.89	0.01*	0.31
ASAS	86.69 (14.02)	72.81 (20.44)	72.19 (17.15)	79.12 (24.44)	86.94 (12.97)	86.94 (11.03)	87.00 (13.29)	82.94 (19.71)	>0.99	0.07	0.10	0.59
SRS	97.88 (15.63)	85.19 (14.58)	80.69 (14.69)	82.12 (15.40)	91.62 (16.62)	87.06 (18.40)	83.25 (15.71)	84.94 (17.59)	0.32	0.79	<0.001***	0.12
Stroop test (RT)												
Test 1	19.65 (4.85)	17.15 (7.64)	15.94 (8.05)	14.80 (6.33)	21.55 (10.07)	16.87 (5.81)	16.36 (5.68)	14.98 (5.85)	0.84	0.84	<0.001***	0.34
Test 2	24.58 (13.30)	20.41 (8.73)	18.00 (8.90)	16.56 (6.69)	21.21 (7.18)	18.24 (6.69)	18.08 (6.61)	16.72 (5.41)	0.69	0.43	<0.001***	0.04*
Test 3	33.25 (16.47)	26.43 (11.04)	23.73 (11.05)	23.47 (9.79)	33.74 (10.95)	26.97 (8.61)	27.69 (12.89)	26.06 (11.94)	0.44	0.78	<0.001***	0.34
Trail Making Test (s)												
Test 1	16.18 (15.73)	15.49 (27.99)	9.95 (9.52)	9.95 (9.65)	13.99 (9.45)	8.73 (3.03)	6.55 (2.13)	7.07 (2.81)	0.72	0.23	<0.001***	0.93
Test 2	20.92 (19.35)	13.12 (10.09)	12.60 (10.54)	10.52 (8.38)	12.37 (4.16)	11.82 (6.07)	9.18 (5.04)	8.57 (3.45)	0.76	0.17	<0.001***	0.04*
Test 3	63.30 (55.89)	41.09 (30.78)	34.50 (17.78)	31.20 (13.14)	42.95 (13.95)	34.00 (13.27)	29.59 (9.02)	28.73 (10.37)	0.84	0.20	<0.001***	0.04*
Digit Span Test												
Score (forward)	11.19 (1.87)	11.62 (2.00)	12.38 (1.54)	12.38 (1.75)	10.44 (2.39)	11.00 (2.45)	11.38 (2.36)	11.62 (2.00)	0.36	0.19	<0.001***	0.82
Score (backward)	6.75 (3.13)	6.38 (3.28)	7.19 (3.06)	7.56 (2.94)	7.94 (3.26)	8.31 (4.04)	8.19 (4.31)	8.62 (3.46)	0.29	0.09	0.0168*	0.55
Length (forward)	98.62 (22.73)	89.50 (17.95)	88.94 (13.61)	77.38 (22.93)	99.07 (39.81)	81.81 (19.52)	85.50 (13.56)	81.94 (19.21)	0.59	0.71	<0.001***	0.60
Length (backward)	111.75 (79.18)	82.88 (75.31)	107.94 (60.87)	119.88 (56.17)	157.93 (137.20)	116.56 (94.32)	107.44 (83.18)	127.00 (71.56)	0.37	0.37	0.65	0.07
Verbal Fluency Test												
Score at 30 s	27.25 (7.22)	31.75 (7.39)	30.62 (8.32)	29.25 (7.95)	27.19 (8.34)	28.81 (9.04)	30.25 (8.95)	29.81 (10.28)	0.87	0.95	0.0402*	0.52
Score at 60 s	39.00 (10.56)	44.56 (11.49)	43.00 (11.79)	41.25 (9.43)	38.69 (14.39)	38.94 (12.89)	42.44 (15.16)	39.62 (16.14)	0.79	0.92	0.22	0.90
CGI												
Severity	5.19 (0.98)	3.81 (1.28)	3.31 (1.20)	3.69 (0.87)	4.62 (0.96)	4.31 (0.48)	3.81 (0.83)	4.19 (0.66)	0.12	0.43	<0.001***	0.02*
Improvement	4.00 (0.00)	1.88 (0.62)	1.81 (0.75)	2.00 (1.03)	4.00 (0.00)	3.94 (0.25)	4.00 (0.00)	3.88 (0.50)	>0.99	<0.001***	<0.001***	<0.001***
Efficacy	1.62 (3.59)	1.31 (2.02)	0.06 (0.25)	0.12 (0.34)	1.56 (3.58)	1.62 (4.44)	0.06 (0.25)	0.06 (0.25)	0.98	0.83	0.002**	0.93
Total	10.81 (3.99)	7.00 (2.37)	5.19 (1.83)	5.81 (1.56)	10.19 (4.17)	9.88 (4.57)	7.88 (0.81)	8.12 (0.96)	0.15	0.006**	<0.001***	0.04*

AQ, Autism Spectrum Quotient Adolescent Version; ASAS, Australian Scale for Asperger’s Syndrome; CARS, Childhood Autism Rating Scale; CGI, clinical global impression; RT, reaction time: s, seconds; SD, standard deviation; SRS, Social Responsiveness Scale.

a Time point: T_1_, baseline; T_2_, immediate after treatment; T_3_, 1 month after treatment; T_4_, 3 months after treatment.

*P < 0.05; **P < 0.01; ***P < 0.001.

### Comparison of CARS score in the study period

Our results revealed a statistically significant improvement (*P* < 0.001) in the severity of clinical symptoms of ASD, including relating with people, emotional response, object use, adaptation to change, listening response, fear of nervousness, verbal communication, level of consistency of intellectual response and general impression before and immediately after TPS. The average CARS score changed from 30.81 (SD: 5.91) to 23.56 (SD: 6.5) (*P* < 0.001). Among all the communication and sensory improvements relating to people, emotional response, adaptation to change, fear of nervousness, verbal communication and general improvement continued to be statistically significant at 3-month F/U (all *P* < 0.05). Only object use and listening response became non-significant at 1- and 3-month F/Us (*P* > 0.05), and the TPS effects on the level and consistency of intellectual response were reduced at 1-month F/U (*P* = 0.048) and became non-significant at 3-month F/U (*P* = 0.173).

Notably, TPS seems to have a longer-term effect on taste, smell and touch response and use of non-verbal communication, as this sensory and communication response were non-significant upon completion of 2-week TPS intervention, but effects became statistically significant at 1- and 3-month F/Us (*P*-values changed from 0.069 to 0.044 after TPS to *P* = 0.034 at 1- and 3-month F/U, respectively). *P*-values changed from 0.069 to 0.048 immediately after TPS and reduced further to 0.045 at 1- and 3-month F/Us for non-verbal communication. In summary, there was a 24% reduction in the average total CARS score [from 30.81 (SD: 5.91) at baseline to 23.44 (SD: 5.66)] after 2 weeks of TPS. Effects were sustained at 1-month F/U [mean CARS score of 23.44 (SD: 7.2)] and at 3-month F/U [mean CARS score of 23.56 (SD: 6.5)]. In particular, the effects of TPS on the total CARS score were sustainable and statistically significant immediately after TPS and at 1- and 3-month F/Us (*P* < 0.05) (Tables 3 and 4).

**Table 3:** Comparison of CARS results before and after transcranial pulse stimulation treatment and 1- and 3-month follow-ups

CARS items		3-month follow-up		1-month follow-up		After TPS		Before TPS		Before versus after TPS	Before versus 1 month	Before versus 3 months
		*n* = 16	%	*n* = 16	%	*n* = 16	%	*n* = 16	%	*P*	*P*	*P*
**Relating to people**										0.015*	<0.001***	<0.001***
	Normal	5	31.3	7	43.8	5	31.3	1	6.3			
	Mild	10	62.5	8	50.0	9	56.3	10	62.5			
	Moderate	1	6.3	1	6.3	2	12.5	4	25.0			
	Severe	0	0	0	0	0	0	1	6.3			
**Imitation**										0.027*	0.007**	0.136
	Normal	7	43.8	11	68.8	8	50	5	31.3			
	Mild	7	43.8	4	25.0	8	50	7	43.8			
	Moderate	2	12.5	1	6.3	0	0	3	18.8			
	Severe	0	0	0	0	0	0	1	6.3			
**Emotional response**										0.007**	0.001**	<0.001***
	Normal	9	56.3	7	43.8	7	43.8	1	6.3			
	Mild	6	37.5	8	50.0	7	43.8	11	68.8			
	Moderate	1	6.3	1	6.3	2	12.5	3	18.8			
	Severe	0	0	0	0	0	0	1	6.3			
**Body use**										0.497	0.791	0.096
	Normal	9	56.3	8	50.0	7	43.8	5	31.3			
	Mild	6	37.5	5	31.3	7	43.8	9	56.3			
	Moderate	1	6.3	2	12.5	2	12.5	2	12.5			
	Severe	0	0	1	6.3	0	0	0	0			
**Object use**										0.029*	0.633	0.096
	Normal	12	75.0	10	62.5	12	75	5	31.3			
	Mild	2	12.5	4	25.0	3	18.8	11	68.8			
	Moderate	2	12.5	1	6.3	1	6.3	0	0			
	Severe	0	0	1	6.3	0	0	0	0			
**Adaptation to change**										0.014*	0.003**	0.003**
	Normal	8	50.0	11	68.8	7	43.8	5	31.3			
	Mild	7	43.8	2	12.5	7	43.8	5	31.3			
	Moderate	1	6.3	3	18.8	2	12.5	5	31.3			
	Severe	0	0	0	0	0	0	1	6.3			
**Visual response**										0.136	0.089	0.027*
	Normal	12	75.0	12	75.0	10	62.5	7	43.8			
	Mild	4	25.0	3	18.8	5	31.3	6	37.5			
	Moderate	0	0	1	6.3	1	6.3	3	18.8			
	Severe	0	0	0	0	0	0	0	0			
**Listening response**										0.014*	0.089	0.111
	Normal	9	56.3	10	62.5	9	56.3	5	31.3			
	Mild	5	31.3	4	25.0	6	37.5	7	43.8			
	Moderate	2	12.5	2	12.5	1	6.3	4	25.0			
	Severe	0	0	0	0	0	0	0	0			

CARS, Childhood Autism Rating Scale; SD, standard deviation; TPS, transcranial pulse stimulation.

*P < 0.05; **P < 0.01; ***P < 0.001.

**Table 4:** Comparison of CARS results before and after transcranial pulse stimulation treatment and 1- and 3-month follow-ups (cont’d)

CARS items		3-month follow-up		1-month follow-up		After TPS		Before TPS		Before versus after TPS	Before versus 1 month	Before versus 3 months
		*n* = 16	%	*n* = 16	%	*n* = 16	%	*n* = 16	%	*P*	*P*	*P*
**Taste, smell and touch response and use**										0.069	0.044*	0.034*
	Normal	8	50.0	11	68.8	7	43.8	6	37.5			
	Mild	7	43.8	3	18.8	7	43.8	3	18.8			
	Moderate	1	6.3	2	12.5	2	12.5	6	37.5			
	Severe	0	0	0	0	0	0	1	6.3			
**Fear or nervousness**										0.002**	0.005**	0.002**
	Normal	8	50.0	7	43.8	9	56.3	2	12.5			
	Mild	6	37.5	7	43.8	5	31.3	7	43.8			
	Moderate	2	12.5	2	12.5	2	12.5	4	25.0			
	Severe	0	0	0	0	0	0	3	18.8			
**Verbal communication**										<0.001***	0.003**	<0.001***
	Normal	6	37.5	4	25.0	7	43.8	1	6.3			
	Mild	9	56.3	11	68.8	9	56.3	6	37.5			
	Moderate	1	6.3	1	6.3	0	0	9	56.3			
	Severe	0	0	0	0	0	0	0	0			
**Non-verbal communication**										0.069	0.048*	0.045*
	Normal	7	43.8	6	37.5	5	31.3	3	18.8			
	Mild	8	50.0	8	50.0	10	62.5	8	50.0			
	Moderate	1	6.3	2	12.5	1	6.3	4	25.0			
	Severe	0	0	0	0	0	0	1	6.3			
**Activity level**										0.669	0.751	1.00
	Normal	8	50.0	8	50.0	9	56.3	8	50.0			
	Mild	5	31.3	6	37.5	4	25.0	5	31.3			
	Moderate	3	18.8	2	12.5	3	18.8	3	18.8			
	Severe	0	0	0	0	0	0	0	0			
**Level and consistency of intellectual response**										0.015*	0.048*	0.173
	Normal	8	50.0	9	56.3	9	56.3	6	37.5			
	Mild	6	37.5	6	37.5	7	43.8	5	31.3			
	Moderate	2	12.5	1	6.3	0	0	5	31.3			
	Severe	0	0	0	0	0	0	0	0			
**General impression**										0.002**	0.015*	0.020*
	Normal	7	43.8	11	68.8	10	62.5	5	31.3			
	Mild	9	56.3	4	25.0	6	37.5	8	50.0			
	Moderate	0	0	1	6.3	0	0	3	18.8			
	Severe	0	0	0	0	0	0	0	0			
**Total CARS score**	Mean (SD)	23.56 (6.50)		23.44 (7.12)		23.44 (5.66)		30.81 (5.91)		<0.001***	0.002**	<0.001***

CARS, Childhood Autism Rating Scale; SD, standard deviation; TPS, transcranial pulse stimulation.

*P < 0.05; **P < 0.01; ***P < 0.001.

Besides, we also reported the group comparison of the improvements in outcome measurements across different time points baseline (T_1_), immediately after 2-week TPS intervention (T_2_), post-stimulation at 1 month (T_3_) and post-stimulation at 3 months (T_4_). CARS, CGI improvement and overall CGI were significantly different between the TPS and sham TPS groups immediately after TPS intervention, and the effects were sustained at 1- and 3-month F/Us, compared with the baseline measurements (all *P* < 0.05). The effect sizes of CARS (*d* = 0.83–0.95) and CGI improvement (*d* = 4.12–4.37) were large to medium immediately after TPS intervention and at 1-month post-stimulation, but effects became small at the 3-month post-stimulation (*d* = 2.31). However, the effects on overall CGI were medium to large (*d* = 0.73–0.91) and sustained at 1- and 3-month F/Us (Tables 5 and 6).

**Table 5:** Comparing changes in outcomes from baseline to immediate after treatment and 1- and 3-month follow-ups

	TPS group	Sham TPS	*Post hoc*	ES
Outcome	Mean (SD)	Mean (SD)	*P*	*d*
T_2_–T_1_				
CAR	−7.38 (5.44)	−1.94 (6.65)	0.03*	−0.9
Stroop test 2 reaction time	−4.17 (7.03)	−2.97 (3.90)	0.94	−0.21
TMT2	−7.81 (11.57)	−0.55 (4.90)	0.44	−0.82
TMT3	−22.21 (36.43)	−8.96 (10.85)	>0.99	−0.49
CGI (severity)	−1.38 (1.63)	−0.31 (0.87)	0.10	−0.81
CGI (improvement)	−2.12 (0.62)	−0.06 (0.25)	<0.001***	−4.37
CGI total	−3.81 (3.41)	−0.31 (5.86)	0.004**	−0.73
T_3_–T_1_				
CARS	−7.38 (7.76)	−1.19 (7.17)	0.047*	−0.83
Stroop test 2 reaction time	−6.58 (10.67)	−3.13 (2.78)	0.56	−0.44
TMT2	−8.33 (13.71)	−3.18 (5.00)	0.87	−0.5
TMT3	−28.81 (45.79)	−13.36 (11.19)	>0.99	−0.46
CGI (severity)	−1.88 (1.36)	−0.81 (0.91)	0.10	−0.92
CGI (improvement)	−2.19 (0.75)	0.00 (0.00)	<0.001***	−4.12
CGI total	−5.62 (3.28)	−2.31 (3.94)	0.003**	−0.91
T_4_–T_1_				
CARS	−7.25 (5.45)	−2.06 (5.50)	0.049*	−0.95
Stroop test 2 reaction time	−8.02 (10.66)	−4.49 (2.81)	0.77	−0.45
TMT2	−10.41 (13.48)	−3.80 (4.42)	0.58	−0.66
TMT3	−32.10 (46.57)	−14.22 (10.42)	>0.99	−0.53
CGI (severity)	−1.50 (1.21)	−0.44 (1.15)	0.10	−0.9
CGI (improvement)	−2.00 (1.03)	−0.12 (0.50)	<0.001***	−2.31
CGI total	−5.00 (3.79)	−2.06 (4.27)	0.004**	−0.73

Time point: T_1_, baseline; T_2_, immediate after treatment; T_3_, 1 month after treatment; T_4_, 3 months after treatment.

CARS, Childhood Autism Rating Scale; CGI, clinical global impression; ES, effect size; SD, standard deviation; TMT, Trail Making Tests 2 and 3; TPS, transcranial pulse stimulation.

*P < 0.05; **P < 0.01; ***P < 0.001.

**Table 6:** Comparing changes in outcomes from post-treatment to 1- and 3-month follow-ups

	TPS group	Sham TPS	*Post hoc*	ES
Outcome	Mean (SD)	Mean (SD)	*P*	*d*
T_3_–T_2_				
CARS	0.00 (5.74)	0.75 (5.27)	0.89	−0.14
Stroop test 2 reaction time	−2.41 (4.31)	−0.16 (3.25)	0.37	−0.59
TMT2	−0.52 (5.92)	−2.63 (5.56)	0.44	0.37
TMT3	−6.59 (17.06)	−4.40 (5.88)	>0.99	−0.17
CGI (severity)	−0.50 (1.41)	−0.50 (0.73)	0.95	0
CGI (improvement)	−0.06 (0.93)	0.06 (0.25)	0.52	−0.18
CGI total	−1.81 (2.40)	−2.00 (4.37)	0.68	0.05
T_4_–T_2_				
CARS	0.12 (4.29)	−0.12 (4.27)	0.89	0.06
Stroop test 2 reaction time	−3.85 (4.83)	−1.52 (3.34)	0.47	−0.56
TMT2	−2.60 (2.95)	−3.25 (5.77)	0.44	0.14
TMT3	−9.89 (20.05)	−5.26 (10.19)	>0.99	−0.29
CGI (severity)	−0.12 (1.41)	−0.12 (0.72)	0.95	0
CGI (improvement)	0.12 (0.96)	−0.06 (0.57)	0.52	0.24
CGI total	−1.19 (2.20)	−1.75 (5.09)	0.68	0.14
T_4_–T_3_				
CARS	0.12 (5.25)	−0.88 (7.16)	0.89	0.16
Stroop test 2 reaction time	−1.44 (3.76)	−1.36 (2.32)	0.56	−0.03
TMT2	−2.08 (4.71)	−0.61 (5.09)	0.58	−0.3
TMT3	−3.30 (9.84)	−0.86 (7.90)	>0.99	−0.27
CGI (severity)	0.38 (1.02)	0.38 (0.62)	0.95	0
CGI (improvement)	0.19 (1.11)	−0.12 (0.50)	0.23	0.36
CGI total	0.62 (1.59)	0.25 (1.06)	0.68	0.28

Time point: T_1_, baseline; T_2_, immediate after treatment; T_3_, 1 month after treatment; T_4_, 3 months after treatment.

CARS, Childhood Autism Rating Scale; CGI, clinical global impression; ES, effect size; SD, standard deviation; TMT, Trail Making Tests 2 and 3; TPS, transcranial pulse stimulation.

### Adverse events

An adverse event checklist was used to monitor any adverse events during and after TPS administration on all participants. Approximately 1/3 of participants (*n* = 5) in the verum TPS group reported transient headaches during the stimulation, with a numeral pain score of 3–5 out of 10. The headache subsided immediately after the TPS session, requiring no pain analgesics. The sham TPS group reported no adverse events. No parents reported any somatic discomfort when participants returned home throughout the intervention period in this trial.

### Blinding

This study included 32 participants, with 16 participants each in the TPS and sham TPS group. All the participants in the verum TPS group correctly identified their treatment allocation; however, seven participants (43.75%) in the sham TPS group guessed the wrong treatment allocation. This indicates that the blinding procedure was effective.

## Discussion

To our best knowledge, this is the first RCT evaluating the effects of TPS in the treatment of core symptoms of ASD worldwide. Specifically, this study aimed to evaluate the efficacy of TPS in 32 young adolescents diagnosed with ASD. We hypothesized that TPS on the rTPJ will cause a 50% improvement in core symptoms of ASD. Our results revealed that TPS successfully reduced 24% of the ASD core symptoms in this study. Notably, CARS was filled in by parents, but the CGI instruments were rated by a licensed mental health professional who objectively assessed each participant’s improvements across different time points in this study. Compared with the CARS score, CGI total score has a 53.7% reduction [from 10.81 (SD: 3.99) at baseline to 5.81 (SD: 1.56) at 3-month F/U] on ASD core symptoms in the verum TPS group. In particular, both parents and mental health expert have a convergent consensus that TPS effectively treated most but not all core symptoms of ASD. Our results are highly encouraging. This is the first study evaluating the effects of TPS on CARS; thus, a comparison with previous studies using other NIBS such as rTMS and transcranial direct current stimulation (tDCS) on ASD is not possible, as the mechanism and mode of administration between TPS and rTMS/tDCS are different.

Some of the core symptoms of ASD, such as body use, listening response, activity intellectual response level and consistency were insignificant after TPS and at 1- and 3-month F/Us in the CARS score. We speculate this phenomenon for several reasons. First, all participants were enrolled in mainstream schools in this trial, and they were expected to actively participate in physical exercise classes and other extra-curricular activities, as assigned by schools. Additionally, out of 32 participants, we had five high-functioning participants and one participant had Asperger’s syndrome with ASD diagnosis in the verum TPS group. These high-functioning participants had less severe symptomatology than other participants who arguably had more severe behavioural and social communication deficits in our sample. Hence, these high-functioning participants may not have notable changes after the TPS intervention. Our speculation is echoed by Casanova *et al*.^38^ Second, our sample comprised participants aged 12–17 years old, and individuals with this age range have passed the age of language improvement.^26^ In particular, brain stimulation effects of TPS on intellectual response among ASD adolescents are least likely to be sustainable compared with toddlers and teenagers aged 3–10. Our *post hoc* analyses revealed that participants in the verum TPS group had a statistically significant reduction in the average CARS score from 30.81 (5.91) at baseline to 23.56 (6.50) at 3-month post-stimulation, compared with the CARS score of 27.94 (7.05) to 25.88 (5.43) in the sham TPS group (*P* = 0.049, *d* = −0.95) regardless of the ASD severity (Table 3). We consider these significant changes in the CARS score in the verum TPS group to be highly encouraging. Notably, placebo effects are found in the sham TPS group but are not statistically significant.

This study included participants aged 12–17 years old, with a mean age of 13.5 (2.03) and 12.81 (1.83) years for the verum TPS and sham TPS groups, respectively. Our sample is relatively young, unlike the other TPS studies. We selected a young cohort in this study intentionally based on the principle of brain plasticity theory. Intervention at an earlier age in neurodevelopmental disorder should have a better treatment outcome than intervening at an older age.^39^ Using a narrow age range in this study allowed us to have a homogenous sample, thereby alleviating the age factor difference that may contribute to different response to TPS intervention.^40^ A similar double-blind RCT^41^ used tDCS on six adults evaluating its effects on social cognition and social skills. This study administered tDCS on the same target brain region (rTPJ). Results revealed that participants had a significantly higher score on the verbal fluency (VF) test in the verum tDCS group compared with the sham tDCS group. Our results echoed Wilson’s study^41^ that revealed a significant time effect (*P* = 0.04) on VF test score at 30 s in the verum TPS group compared with the sham TPS group. However, we need to interpret Wilson’s study findings with caution due to its small sample size and wide age range of adult samples (18–58 years). Another recent NIBS single-blind RCT^42^ used tDCS to evaluate the WM on 12 high-functioning adults with ASD. Bifrontal stimulation was applied to the left and right dorsolateral prefrontal cortex. Results revealed significant improvement in overall WM in the verum tDCS group compared with the sham tDCS. Our study findings were similar to Van Steenburgh’s^42^ where the Digit Span Test score (forward and backward) had a significant improvement and time effect (*P* < 0.001 and *P* = 0.0168, respectively) on the verum TPS group. VF and WM improvement in our study has an important indication that brain stimulation of the rTPJ is also effective to improve social cognition and social and communication skills deficits in ASD.

In summary, our current study has a clear answer to our research question that TPS on rTPJ is a safe, novel, effective treatment of ASD core symptoms, including executive functioning, WM and social cognition.

### Limitations of the study

This study revealed that TPS is effective in treating some core symptoms of ASD; however; some limitations should be addressed. First, we confined our sample to be diagnosed with ASD. rTMS has proven effective in the treatment of ASD symptoms in toddlers aged 4–10 years^25^ or 5–8 years old,^24^ and both studies focused on the left dorsolateral prefrontal cortex. Other studies focus on both left and right dorsolateral prefrontal cortex,^42^ and one TMS study focused on the left inferior frontal gyrus.^26^ Seemingly, there is a lack of consensus in existing brain stimulation studies on which brain treatment regions can produce both short- and long-sustainable effects on ASD symptoms. Therefore, a multi-site international collaboration investigating the effects of TPS on different age groups is warranted. Second, some participants’ parents suffer from other psychiatric disorders (e.g. anxiety disorder), and they might have high levels of expectations for their children to have prompt recovery after TPS treatment. It possibly explains why some participants in the sham TPS group have improved CARS scores after stimulation. Future replication of this study should exclude both parents and children who are co-morbid with other psychiatric disorders, other than ASD to filter out the placebo effects.

## Conclusion

This study concluded that six TPS sessions over rTPJ are effective and well tolerated to treat the core symptoms of young adolescents with ASD. It is recommended that future replication of similar studies should include a larger sample derived from multi-nations to determine whether TPS could be considered as a top-on treatment for ASD in neuropsychiatry shortly.

## Data Availability

Anonymized data are available on reasonable request from the corresponding author.
